# Mapping of Recurrence Sites Following Adjuvant or Salvage Radiotherapy for Prostate Cancer Patients

**DOI:** 10.3389/fonc.2021.787347

**Published:** 2022-01-05

**Authors:** Ana Gonzalez-Moya, Stéphane Supiot, Valérie Seegers, Thibaut Lizée, Florence Legouté, Tanguy Perennec, Gilles Calais

**Affiliations:** ^1^ Department of Radiation Oncology, Institut de Cancérologie de l’Ouest, Angers, France; ^2^ Department of Radiation Oncology, Institut de Cancérologie de l’Ouest, Nantes, France; ^3^ Department of Medical Biostatistics, Institut de Cancérologie de l’Ouest, Angers, France; ^4^ Department of Radiation Oncology, Jean Bernard Center, Inter-Regional Institute of Oncology, Le Mans, France; ^5^ Department of Radiation Oncology, Hospital Center, La Roche sur Yon, France; ^6^ Department of Radiation Oncology, University Hospital Center, Tours, France

**Keywords:** pattern of recurrence, adjuvant/salvage radiotherapy, prostate cancer, pelvic radiotherapy, prostate bed radiotherapy

## Abstract

**Introduction:**

Although salvage and adjuvant radiotherapy (RT) are effective in prostate cancer (PC) patients, 30%–40% of men will have disease progression. The objective was to describe the pattern of recurrence in PC patients with biochemical failure (BF) following postoperative RT.

**Methods:**

We retrospectively analyzed 935 PC patients treated from 2009 to 2019 with adjuvant or salvage RT at the Institut de Cancérologie de l’Ouest. Of these, 205 (22%) developed BF of whom 166 underwent imaging. Patients with identified radiologic failure prior any specific treatment were included to determine the site of relapse categorized as local (L)-only, locoregional (LR), or metastatic (M) recurrence. Main disease characteristics and RT fields were examined in relation to sites of recurrence.

**Results:**

One hundred forty-one patients were identified with 244 sites of failure on imaging. Of these, 108 patients had received RT to the PB alone and 33 RT to the PB and pelvic lymph nodes (PB+PLN). Androgen-deprivation therapy was used concomitantly in 50 patients (35%). The median PSA at imaging was 1.6 ng/ml (range, 0–86.7). In all, 74 patients (52%) had M disease (44% in the PB group and 79% in the PB+PLN group), 61 (43%) had LR failure (52% in the PB alone group and 15% in the PB+PLN group), and six (4%) had L-only failure, at a median of 26.7 months (range, 5–110.3) from RT. Metastases were in extra-pelvic LN (37 (15%)), bones (66 (27%)), and visceral organs (eight (3%)). Fifty-three (48%) of the pelvic LN failures in the PB group would have been encompassed by standard PLN RT volume.

**Conclusion:**

We found that most patients evaluated for BF after postoperative RT recurred outside the RT field. Isolated pelvic nodal failure was rare in those receiving RT to the PB+PLN but accounted for half of failures in those receiving PB alone RT. Imaging directed salvage treatment could be helpful to personalize radiation therapy plan.

## Introduction

Approximately 20% to 40% of patients treated with radical prostatectomy (RP) for prostate cancer (PC) will present biochemical recurrence (BCR) defined by an increase of the prostate-specific antigen (PSA) level on two or more consecutive determinations or a persistently rising PSA greater than 0.2 ng/ml after RP (1, 2). Prostate bed (PB) radiotherapy (RT) is the standard postoperative treatment after RP for tumors with high-risk features or persistent PSA or for salvage treatment in case of BCR (3). Whether RT should be limited to the PB or should include pelvic lymph nodes (PLN) is still controversial. The NRG Oncology/RTOG 0534 SPPORT trial found an improvement in biochemical control, but there is lack of benefit in progression-free survival (PFS) in two trials (RTOG 9413 and GETUG01) (4–6). Despite salvage treatment, rates of recurrence following postoperative RT remain high: almost 30%–40% at 5–10 years (7).

Accurate estimation of the relapse site after RP is important to choose the correct salvage therapy and to ensure sufficient dose and coverage by RT when indicated. Recent evidence suggests improved metastasis-free survival in men receiving salvage RT with PSA level ≤0.5 ng/ml (8, 9). However, the rate of detection of metastases from computed tomography (CT), magnetic resonance imaging (MRI), and bone scintigraphy is poor in this setting (10). As such, RT is usually decided without histological or imaging proof of recurrence, and the defined target volumes are usually drawn in the absence of visible disease. Some temporal and biological characteristics (Gleason score, PSA doubling time (PSADT), and time to relapse after local treatment) are predictive of survival and response to complementary therapies (11). However, it cannot distinguish between local, regional, or systemic recurrence; nevertheless, this information is essential for further management. Positron emission tomography (PET)-CT use, with radioactive tracers including fluorine 18 (^18^F)-choline, ^18^F-fluciclovine, and gallium 68 (^68^Ga) or ^18^F-prostate-specific membrane antigen (PSMA), permits recurrent disease detection, even at low PSA levels. PET-CT can be useful for restaging PC and guiding treatment delivery (12, 13). Several imaging studies in men with PSA failure reported sites of post-RT recurrence disease (14, 15). However, only diagnostic performance and impact of PET-CT on RT planning were assessed. Other limitations were observed: inconsistent descriptions of anatomic relapse patterns and several inhomogeneous patient groups (wide range of PSA values and clinical states).

Patterns of failure after postoperative RT are critical to better understand how to improve patient outcomes. Therefore, the aim of this study was to describe pattern of failure in patients who received adjuvant or salvage RT and experienced biochemical failure (BF). Their relationship to the main predictive clinical factors and to prior RT fields was also studied.

## Materials and Methods

### Patient’s Selection and Treatment

**Figure 1:**
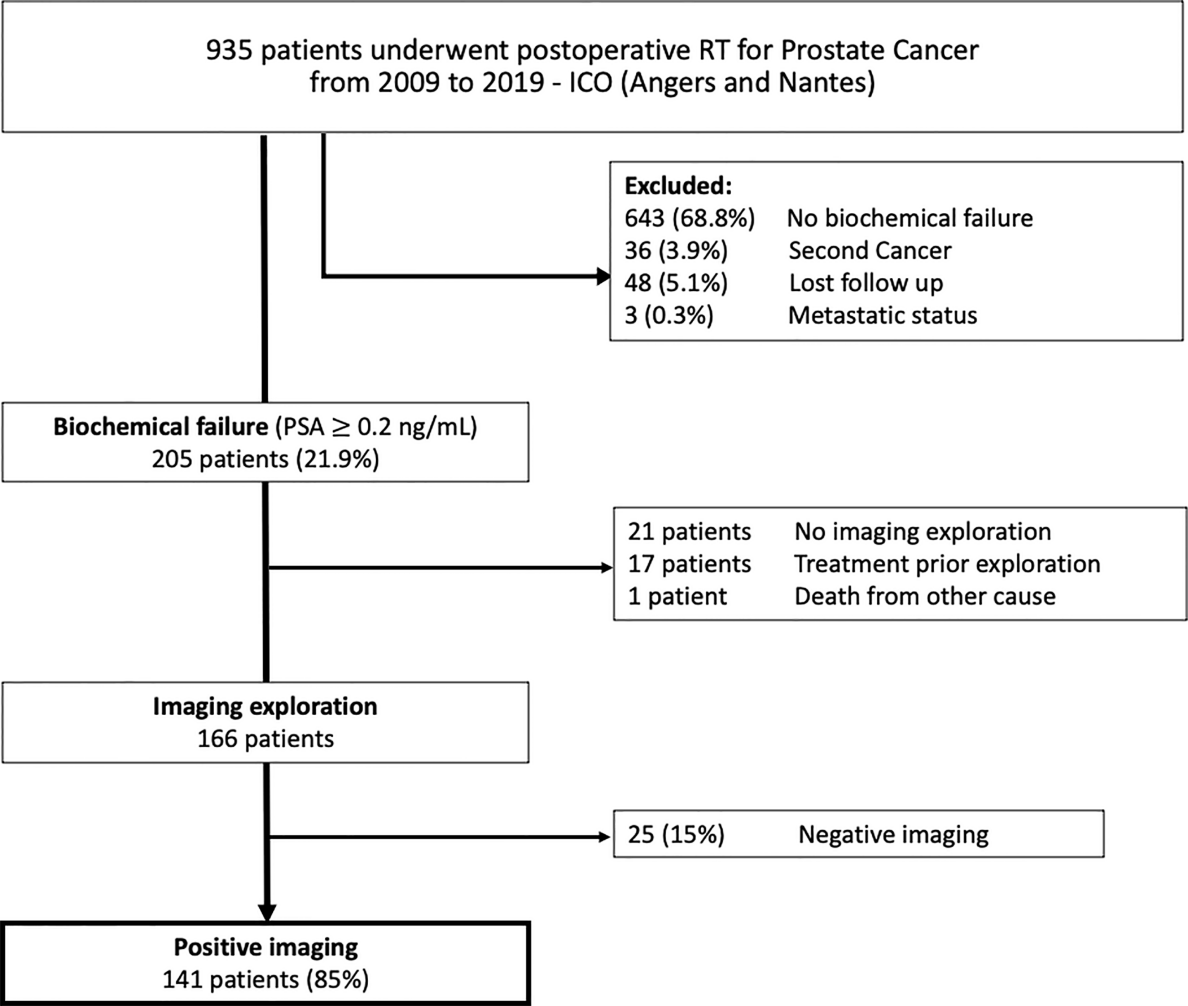
Flow chart of study population. ICO, “Institut de Cancérologie de l’Ouest”; RP, Radical Prostatectomy; RT, radiotherapy.

We retrospectively reviewed medical records of 935 patients with PC treated from 2009 to 2019 with external beam RT after RP at the Institut de Cancérologie de l’Ouest (ICO, Nantes and Angers, France). All patients had pathologic confirmation of PC and had undergone RP, with or without lymphadenectomy. Patients had localized or locally advanced PC with no evidence of disseminated disease. Patients having second cancer were excluded. Of the 935 patients, 205 (22%) developed BF defined as a rising PSA level of ≥0.2 ng/ml after RT. Many of whom (166 patients) underwent imaging study prior any specific treatment. Thirty-nine patients with BF did not undergo imaging: 17 received systemic therapies (including ADT), one patient died of other causes not related to PC and 21 patients had a slow-rising PSA without having a scan performed to date. Patients with radiologic failure prior any specific treatment were included to determine relapse sites; 141 men met the inclusion criteria and formed the cohort study (**Figure 1**). Approval for the study protocol was obtained from the medical research ethics committee (2019/88) before the study was conducted. Written informed consent was obtained from all patients.

Postoperative RT was delivered as adjuvant, for men with adverse pathologic features, or salvage, in the setting of detectable PSA. The clinical target volumes (CTV) were contoured as per departmental protocol based on the international consensus guidelines published (16, 17). The PB CTV included prostatic fossa and seminal vesicle remnants. PLN were also irradiated in men with high-risk clinicopathological features (23%, *n* = 33), and neoadjuvant or concurrent ADT was prescribed in 50 patients (35%) at the radiation oncologist’s discretion. The PLN CTV included obturator, external iliac, internal iliac, presacral and common iliac nodes, using the vascular structures, up to a level corresponding to the top of L5-S1. In this study, no patient had extended nodal RT (to the aortic bifurcation) treatment volumes. The planning target volume (PTV) consisted of the CTV plus a uniform 0.5–1.0-cm expansion. The median dose delivered to the PB PTV was 66 Gy (range, 60–74) delivered in daily 2 Gy fractions. If PLN were treated, they received a median dose of 46 Gy (range, 44–59.4). In seven patients with biopsy or imaging proven gross recurrence, higher doses were delivered to the PB (*n* = 4) or selected PLN (*n* = 3).

### Pattern of Failure Analysis

To detect recurrence patterns in the setting of first-time PSA failure after postoperative RT, several imaging modalities were available: CT and bone scintigraphy, ^18^F-choline PET-CT, or ^68^Ga-PSMA-PET-CT. For patients with identified sites of recurrence, number and location of lesions were documented. Failure sites were categorized as local-only (disease within the prostate fossa only), locoregional (nodal disease within the pelvis ± PB) or metastatic failure (at least one lesion outside the pelvis ± locoregional disease). Metastatic failure includes disease within distant LN (lumbo-aortic, abdominal/retroperitoneal, mediastinal, and supraclavicular LN stations), bone, visceral, or combination of these three subgroups (multisite), with or without synchronous locoregional recurrence. A binary method was used to identify recurrence site (involved or uninvolved), rather than documenting the number of involved lesions within each site. Bilateral lesions were merged to one station. Sites of failure were then analyzed separately in two groups of patients based on the treated RT volume they received: RT to the PB alone or to the PB+PLN. Dose distributions from printed plans were used and sites were characterized as either included (inside the PTV) or not included (outside the PTV) within these fields. Time to recurrence (TTR) was defined from the date of RT to the date of imaging evidence of recurrence. BF after RT was defined as a rising PSA level of ≥0.2 ng/ml. Follow-up was calculated from completion of RT for all patients.

### Statistical Analysis

Descriptive statistics were used to define failure patterns. Continuous variables (quantitative data) were summarized using mean, medians, and interquartile range (IQR): 25th and 75th percentiles and range (minimum and maximum) according to their distribution. Categorical variables (qualitative data) were summarized using frequencies and percentages. An exploratory analysis of recurrence site was performed thanks to RT volume collection: it allows to describe recurrences relative to these anatomic distributions. To test the association of recurrence location with clinical features, data was evaluated by patient (and by lesion). The censored data were summarized using survival function according to Kaplan-Meier method. For exploratory analysis, no correction of the *p*-values was performed for multiple tests. Analysis was undertaken by Biometrics and Statistics unit of the ICO using the R Statistical software (R Core Team – 2019 ^©^).

## Results

### Patients’ Characteristics

Clinical features of the cohort are summarized in **Table 1**. Median age and median PSA at diagnosis was 66 years and 7.7 ng/ml, respectively. Most patients (132/141; 94%) were National Comprehensive Cancer Network (NCCN)–defined high risk, 96% (136/141) had International Society of Urological Pathology (ISUP) grade ≥2 and 62% (88/141) had ≥pT3a stage. Among 141 patients, 77% had received RP with standard LN dissection and 23% had RP alone. Median number of LN removed was 4 (range, 1–31). Following RP, an undetectable PSA was achieved in 94 (67%) patients.

**Table 1:** Baseline characteristics for all patients and subgroups of local-only (L), locoregional (LR) and metastatic (M) sites of recurrences.

Characteristic	Total (n=141)	L (n=6)	LR (n=61)	M (n=74)
Median age at diagnosis, y (range)	66 (43-77)	62 (57 – 70)	65 (50 – 76)	66 (43-77)
PSA value at diagnosis, No. (%)				
<10 ng/ml	99 (70%)	5 (83%)	45 (74%)	49 (66%)
≥10 to <20 ng/ml	36 (26%)	1 (17%)	15 (25%)	20 (27%)
≥20 ng/ml	5 (4%)	0	1 (2%)	4 (5%)
Unknown	1 (1%)	0	0	1 (1%)
ISUP grade, No. (%)				
ISUP 1	5 (4%)	1 (17%)	4 (7%)	0
ISUP 2	50 (35%)	2 (33%)	26 (43%)	22 (30%)
ISUP 3	54 (38%)	3 (50%)	20 (33%)	31 (42%)
ISUP 4	32 (23%)	0	11 (18%)	21 (28%)
Pathologic T-stage, No. (%)				
pT2	53 (38%)	4 (67%)	24 (39%)	25 (34%)
pT3a	47 (33%)	1 (17%)	26 (43%)	20 (27%)
pT3b	40 (28%)	1 (17%)	10 (16%)	29 (39%)
pT4	1 (1%)	0	1 (2%)	0
Pathologic N-stage, No. (%)				
pN1	9 (6%)	0	2 (3%)	7 (9%)
pNx	32 (23%)	3 (50%)	16 (26%)	13 (18%)
Positive Surgical Margins (R1), No. (%)	72 (51%)	3 (50%)	26 (43%)	43 (58%)
Undetectable PSA post-RP, No (%)	94 (67%)	5 (83%)	50 (82%)	39 (53%)
PSADT before RT, No. (%)				
<10 months	62 (44%)	4 (67%)	25 (41%)	33 (45%)
≥10 months	47 (33%)	2 (33%)	23 (38%)	22 (30%)
Unknown	32 (23%)	0	13 (21%)	19 (26%)
PSA level before RT, No. (%)				
<0.2 ng/ml	15 (11%)	1 (17%)	9 (15%)	5 (7%)
≥0.2 to <0.5 ng/ml	76 (54%)	3 (50%)	34 (56%)	39 (53%)
≥0.5 to <1 ng/ml	36 (26%)	2 (33%)	14 (23%)	20 (27%)
≥1 ng/ml	14 (10%)	0	4 (7%)	10 (14%)
Salvage/adjuvant RT field, No. (%)				
PB only	108 (77%)	4 (4%)	56 (52%)	48 (44%)
PB + PLN	33 (23%)	2 (6%)	5 (15%)	26 (79%)
Concurrent ADT, No (%)	50 (35%)	1 (17%)	18 (30%)	31 (42%)
Adjuvant RT, No (%)	17 (12%)	1 (17%)	10 (16%)	6 (8%)
Median time to RT from RP, mo (range)	19.5 (3 – 138)	11 (8 – 63)	22 (4 – 138)	16 (3 – 123)
Median TTR after RT, mo (range)	27.0 (5 – 110)	52 (25– 110)	29.7 (6 – 101)	20.9 (5 – 100)

ADT, Androgen-Deprivation Therapy; BF, Biochemical Failure; ISUP grade, International Society of Urological Pathology grade; L, local-only; LR, locoregional; M, metastatic; mo, month; PB, Prostate Bed; PLN, Pelvic Lymph Nodes; PSA, Prostate Specific Antigen; PSADT, PSA Doubling Time; RP, Radical Prostatectomy; RT, Radiotherapy; TTR, Time to recurrence; y, year.

In our cohort, postoperative RT was completed between January 2009 and November 2019 and involved both three-dimensional (3D) conformal RT (*n* = 61, 43%) or intensity-modulated RT (IMRT) (*n* = 88, 57%) delivered at a median of 19.5 months (range, 3–137.6) following surgery. Sixteen patients (11%) received adjuvant RT, and 125 patients (89%) received salvage RT due to a detectable PSA with a median pre-RT PSA level of 0.3 ng/ml (range, 0–5.4), of whom 58 patients (47%) have PSADT of ≤10 months. The median follow-up was 5.8 years after RT (95% CI: 4.9–6.3).

**Figure 2:**
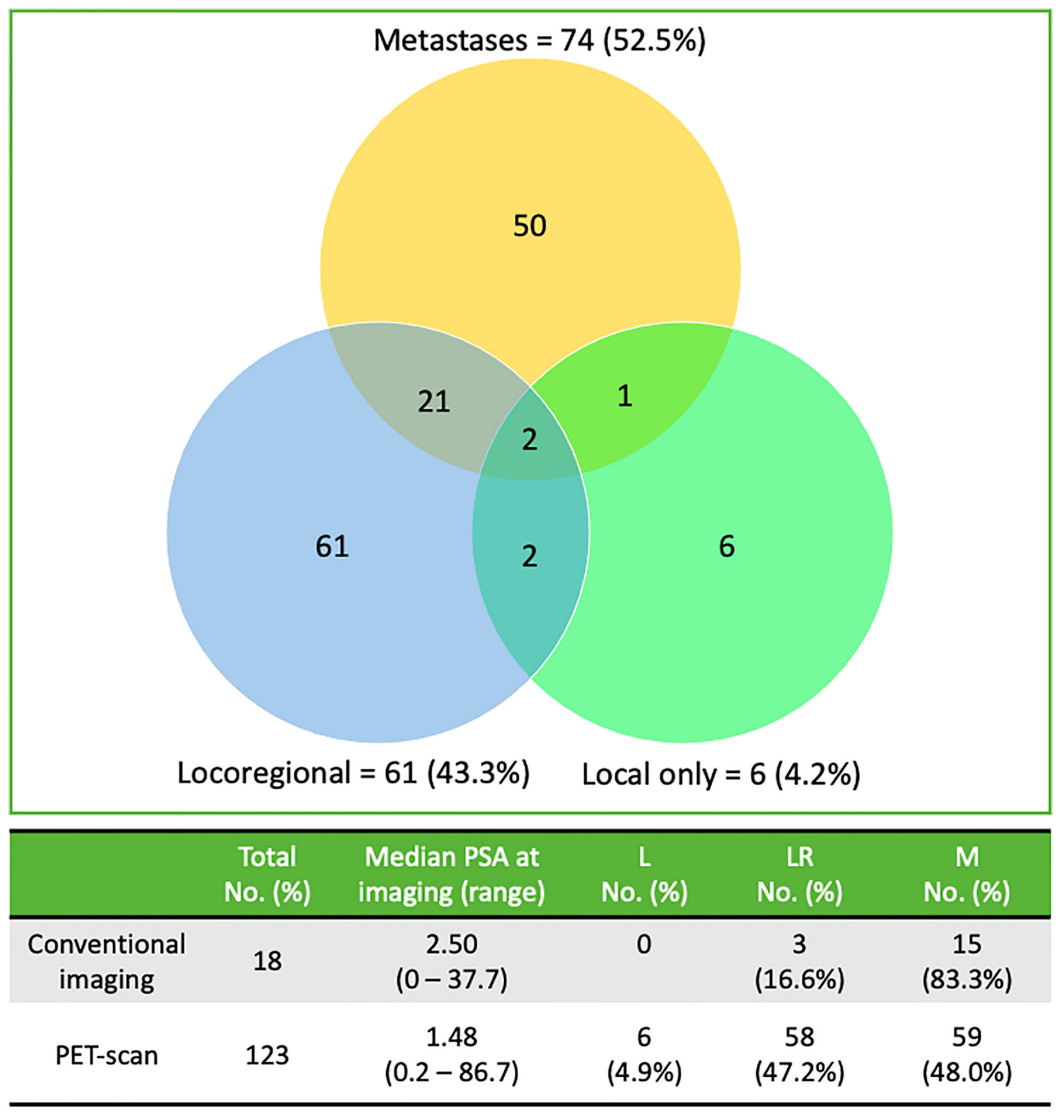
Venn diagram of radiologically patterns of failures’ distribution for the 141 patients and imaging tools (conventional imaging vs PET) used to detect recurrence location. Lesions are categorized as local only (disease within the region of the PB), locoregional (defined as the presence of pelvic LN ± PB) or metastatic failure (at least one distant lesion outside the pelvis ± locoregional disease). LN, Lymph Nodes; PET, positron-emission tomography.

A total of 141 patients had detectable lesions on imaging with 244 sites of failure identified after postoperative RT. From 2012 to 2021, recurrences were detected in 18 patients on conventional imaging (CT and/or bone scan) versus in 123 patients on PET-CT (^18^F-choline (*n* = 99) and ^68^Ga-PSMA (*n* = 24)). The time interval from BCR post-RT (PSA level ≥0.2 ng/ml) to imaging evidence recurrence was 8.5 months (range, 0.1–86.3). Venn diagram to illustrate overlap of the failure sites and imaging tools used to detect recurrence location is shown in **Figure 2**. Among the entire cohort, 74/141 patients (52%) had metastatic disease, 61/141 (43%) had locoregional-only recurrence, and six of 141 (4%) had local-only failure, at a median of 26.7 months (range, 5–110.3) from RT.

Patients with metastatic failure had shorter TTR (median, 20.9 vs. 29.7 and 52 months), more seminal vesical invasion (pT3b stage in 39% vs. 16% and 17%) and positive LN (pN1 in 9% vs. 3% and 0%) than the regional and local ones, respectively. Distant recurrences were higher in patients with ISUP grade of 3 or more than in those with a ISUP grade of 2 or less (52/74 (70%) vs. 22/74 (30%)) and in ≥pT3a patients than in pT2 patients (49/74 (66%) vs. 25/74 (34%)). Only 39/74 patients (53%) had undetectable PSA level after RP versus 55/67 (82%) for locoregional ones. In men with a pre-RT PSA of ≥ 0.5 ng/ml, rate of distant failure was 60% (30/50), compared with 48% (44/91) in those with a pre-RT PSA of <0.5 ng/ml. Patients receiving adjuvant RT (*n* = 17) had a higher proportion of positive surgical margins (R1 in 76.5% vs. 48%), ≥ pT3a stage (88% vs. 58%) and ISUP grade ≥4 (59% vs. 18%) than patients receiving salvage RT (*n* = 124). The rate of positive LN (pN1) after RP did not differ between adjuvant and salvage RT group. As anticipated, patients receiving ADT had also significantly higher risk factors than the other ones. They had a higher proportion of Gleason score ≥8 (34% vs. 15%), PSA before RT >0.5 ng/ml (38% vs. 13%) and shorter TTR (median, 10.8 vs. 23.3 months).

### Pattern of Recurrence Sites

**Figure 3:**
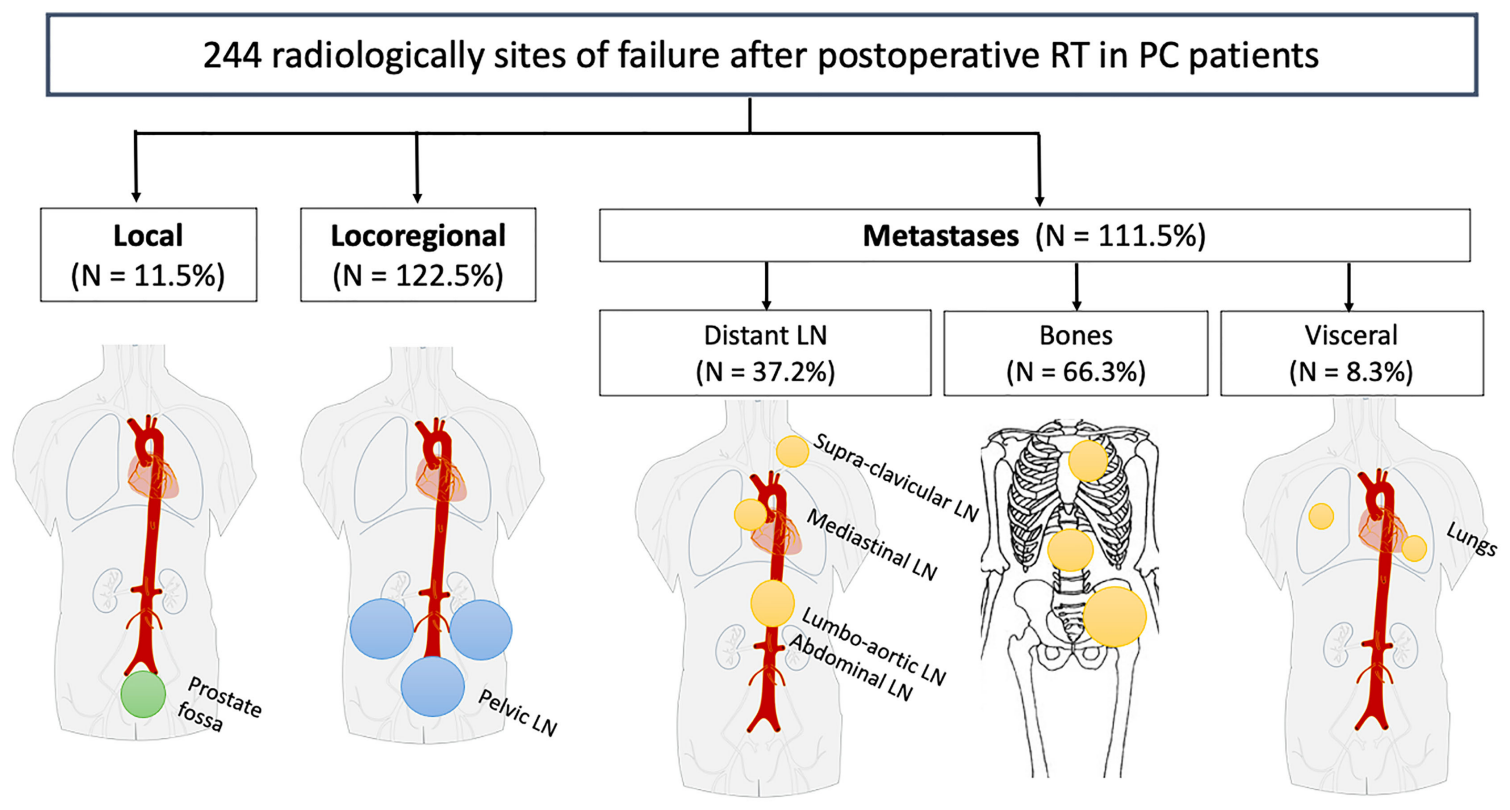
Diagram depicting anatomical sites of recurrence for the 244 radiologically sites of failure after postoperative RT in PC patients. LN, Lymph Nodes; PC, Prostate Cancer; RT, radiotherapy.

Radiologically failure sites for all patients are detailed in **Table 2** with pseudo-anatomical representation in **Figure 3**. In total, 16 (11%) patients had histologic confirmation of their disease. Median PSA at detection of recurrence was 1.6 ng/ml (range, 0–86.7). One patient had distant failure without preceding BF (bone metastases with undetectable PSA level).

**Table 2:** Total distribution of the 244 clinically detectable sites of recurrence.

Site of failure	Total, No. (%)
Local – (T)	11 (5%)
Regional – Pelvic nodes (N)	122 (50%)
Common iliac LN	19 (8%)
External iliac LN	57 (23%)
Pre sacral LN	12 (5%)
Internal iliac and obturator LN	31 (13%)
Peri rectal LN	3 (1%)
Metastatic (M)	111 (45%)
Distant LN (M1a)	37 (15%)
Lumbo-aortic LN	27 (11%)
Abdominal/retroperitoneal LN	6 (2%)
Mediastinal LN	3 (1%)
Sus clavicular LN	1 (0.4%)
Bone (M1b)	66 (27%)
Lung (M1c)	8 (3%)

The total distribution of detectable sites of recurrence for the 141 patients is presented and staged as in the TNM system according to NCCN.

LN, Lymph Nodes; NCCN, National Comprehensive Cancer Network.

Most of the recurrence sites were distant metastases with 74 of the 141 patients (52%) and 111 lesions sites. For these 74 patients, median PSA at the time of imaging was 2.1 ng/ml (range, 0–86.7). Median time to BCR after surgery was 12.8 months (range, 0.7–106.9), and median TTR after RT was 20.9 months (range, 5.0–99.7). Among them, three patients were found to have local recurrence, 22 patients had disease within the pelvis in additional to their distant sites of metastases, and 50 had distant metastases alone. Sites of distant metastases included lumbo-aortic (*n* = 27), abdominal/retroperitoneal (*n* = 6), mediastinal (*n* = 3), and supraclavicular (*n* = 1) LNs, in addition to bone (*n* = 66) and lung (*n* = 8) metastases. Regarding the number of metastatic sites, 24 patients (33%) had a solitary site of metastasis, 27 patients (38%) had two to five sites of disease and 21 patients (29%) had high metastatic burden with ≥ 6 sites. In total, 61 of the 141 patients (43%) presented locoregional recurrences with 122 lesions sites identified within pelvic LN. They were in the external iliac (*n* = 57), internal iliac/obturator (*n* = 31), common iliac (*n* = 19), presacral (*n* = 12), and perirectal (*n* = 3) areas. Two of these 61 patients were found to have a local recurrence in addition to their pelvic nodal disease. Median PSA at the time of imaging was 1.3 ng/ml (range, 0.2–16.9). Median time to BCR after RP was 19.2 months (range, 1.5–135.1), and median TTR after RT was 29.7 months (range, 5.5–100.5). Local failures were rare with 11 lesions in total. Recurrences were local only in six of 141 patients (4%); the other five patients had additional sites of recurrence out of the treated field. Local recurrences occurred at the urethral anastomosis (*n* = 5), followed by the retrovesical area (*n* = 4) and the seminal vesicle bed (*n* = 2). Median PSA at the time of imaging was 1.2 ng/ml (range, 0.4–1.8), and median TTR after RT was 52 months (range, 24.7–110.3).

**Figure 4:**
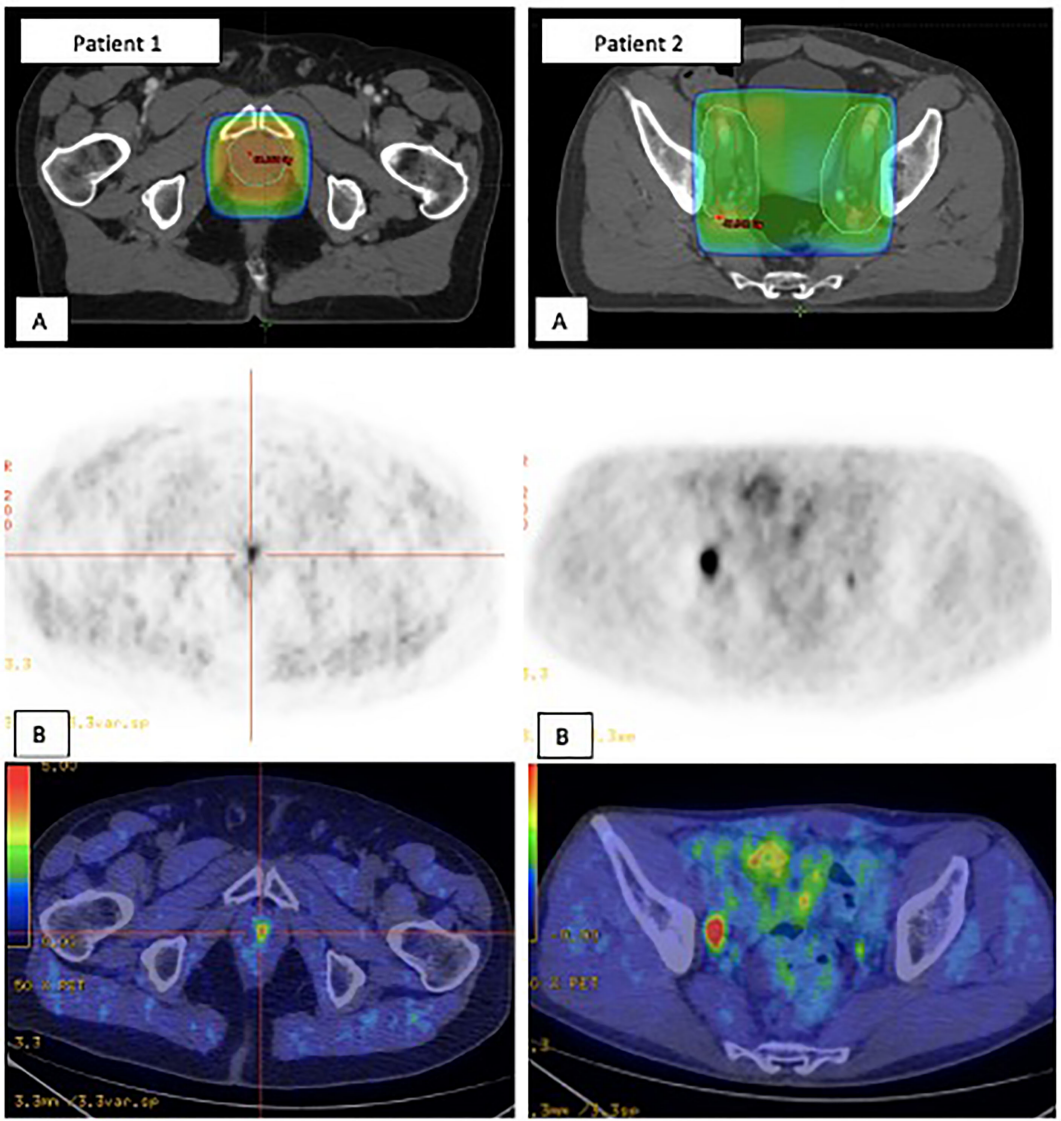
Two patients’ examples of in-field recurrences **(A)** which were positive on Choline PET/CT **(B)**. Patient 1. Urethral anastomosis failure within the PB RT volume. Patient 2. Right iliac extern nodal failure within PLN RT volume. PB, Prostate bed; PET-CT, Positron emission tomography-computed tomography; PLN, Pelvic lymph nodes; RT, Radiotherapy.

Sites of recurrence and their relationship to prior RT planning fields, both for the PB alone and the PB+PLN groups, are summarized in **Table 3**. Of the 108 patients treated to the PB alone, isolated infield failure occurred in 4 patients (4%) and all recurred within the radiation isodose 66 Gy (patient’s example 1 in **Figure 4**). In these group, 104 (96%) failed out of the previous radiation field; half of them (*n* = 56, 52%) had locoregional disease and 48 (44%) had metastatic disease. Notably, 53 of the 122 pelvic nodal recurrences (48%) might have been encompassed by the addition of the standard PLN volume (PTV to L5-S1 fields). In the PB+PLN group, two of 33 patients (6%) had local-only failure. Five of 33 patients (15%) had pelvic nodal failure alone: four were located within the PLN RT field, and one located just outside the RT volumes in the pararectal area. In these group, 26/33 patients (79%) had metastatic disease: none of the 33 patients (27%) had distant LN, 12/33 (40%) had either bony or visceral metastases, and five of 33 (15%) had multisite failure. Five patients had nodal failure both within the pelvis and distant LN: four patients had LN within RT fields and one had presacral LN located just above the RT volume.

**Table 3:** Summary of type and sites of failure relative to prior RT field.

Pattern of failure	All patients No. (%)	PB alone group No. (%)	PB+PLN group No. (%)
Total	141 (100%)	108 (77%)	33 (23%)
Local-only	6 (4%)	4 (4%)	2 (6%)
Locoregional	61 (43%)	56 (52%)	5 (15%)
Metastatic	74 (52%)	48 (44%)	26 (79%)
Distant LN	27 (19%)	18 (17%)	9 (27%)
Bone	37 (26%)	26 (24%)	11 (33%)
Visceral (lungs)	5 (3.5%)	4 (4%)	1 (3%)
Distant Multisite	5 (3.5%)	0	5 (15%)

Sites of failure are summarized for all patients and subgroups of patients based on the prior treated RT volume (PB alone or PB+PLN).

PB, Prostate Bed; PB+PLN, Prostate Bed and Pelvic Lymph Nodes.

In total, 26 patients (18%) relapsed within 1 year, 41 (29%) within the second year, 59 (42%) within 3 to 5 years and 15 (11%) over 6 years after RT. In patients irradiated to the PB alone compared with those treated to the PB+PLN, fraction of the recurrences occurring within 1 and 2 years was approximately two times higher. The fraction of patients with relapse within 3–5 years and over 6 years was similar in both patient groups. Retreatment after postoperative RT was successful in 31% of relapse patients (44/141) with a median follow-up of 47 months (95% CI: 40.4–55.2). At analysis, 96 of 141 patients (68%) had received retreatment for new recurrent disease (82 patients received systemic therapies and only seven received RT to the recurrent site) and 6% of the patients had died of PC.

## Discussion

The current study provides a perspective on recurrence patterns after postoperative RT and their correlation to predictive clinical factors and to prior radiation fields. Pattern failure identification is challenging because it requires long-term follow-up of large numbers of patients. Moreover, many centers use early ADT for PSA failure, which may delay further identification of failure site. To our knowledge, this study included the largest cohort of PC patients to assess failure patterns after postoperative RT. Using conventional or PET-CT imaging techniques, 244 sites of recurrence were identified in 141 patients. Our data support previous findings: in patients with BF after postoperative RT, metastatic disease is the main failure pattern (18–20).

Literature about BF after postoperative RT is limited. Byrne et al. assessed recurrence patterns after PB only and PB+PLN (*n* = 17) postoperative RT in 67 patients who underwent ^68^Ga-PSMA-PET (21). There were distinct failure patterns with local only, nodal only, distant only, and multisite failure of 4%, 66%, 16%, and 14% for the PB alone group compared with 0%, 41%, 41%, and 18% for the PB+PLN group. It should be noticed that this nodal figure includes patients with PLN plus para-aortic and distant LN. Our equivalent figures would be nodal only 69% and 42%, distant only 28% and 36%, and multisite 0% and 15% for the PB and PB+PLN group, respectively, which appear concordant. Recently, Rowe et al. identified 32 patients with BF and ^18^F-PSMA PET-CT avid lesions after postoperative RT to the PB alone and to the PB+PLN (*n* = 6) (22). Seventeen patients (53%) had metastatic disease, eight of 32 (25%) patients had locoregional recurrences, and seven of 32 (22%) had local failure. Jackson et al. have reported among 574 men who received PB only or PB+PLN (*n* = 23) salvage RT after RP, 128 cases with recurrence pattern: distant disease (84%), pelvic nodal (12%), and local failure (5%) (21). Notably, 64% of recurrences were first in bone. Elevated pre-RT PSA levels (0.7; range, 0.4–1.4 ng/ml) in their cohort may have in part contributed to the overall high rate of metastatic progression. Another explanation for the high distant failure could be ADT use to only 25% of men. However, we also find a high rate of metastatic disease in our cohort with 74/141 patients. In addition, bone metastatic disease was found in 66 patients. We also had only 35% of patient receiving ADT during RT, but our pre-RT PSA level was lower (median, 0.3 ng/ml). As shown in recent randomized trials, ADT use should strongly be considered, especially for men with high presalvage RT PSA levels (23). Other measures are needed to decrease rates of distant metastases in addition to ADT and to assess possible benefits of treatment intensification with chemotherapy, second-generation antiandrogens, or other innovative therapies in the context of postoperative RT.

In addition, several imaging studies suggested that the pelvic nodes are also a common site of failure after RP alone (22, 24–26) and after RT to the PB alone (27, 28). In our study, 48% of the sites of pelvic recurrence would be encompassed within standard PLN RT fields. These findings are consistent with previous series (21, 29). Moreover, higher proportion of pelvic node failure after treating the PB alone in our cohort (52%) could justify PLN irradiation. This is also supported by the early outcomes of the NRG Oncology/RTOG 0534 SPPORT trial: whole PLN irradiation after RP and BF improves biochemical control with a low toxicity (8). According to exploratory subgroup analysis, the benefit may be limited to men with a PSA >0.34 ng/ml at the time of salvage treatment. In our study, only 19 patients of 56 patients in the PB alone group with locoregional failure would have met these criteria. Moreover, Brand et al. described the same rate of pelvic nodal failure for patients with PSA above or below 0.34 ng/ml.

Challenge is to better identify patients who may benefit from RT to the PB+PLN. Nomograms might be useful, but tools such as Briganti nomogram are yet to be validated in the postoperative salvage setting (30). Recently, increasing use of new imaging modalities such as ^68^Ga or 18F-PSMA PET-CT in PC patients have improved localization of recurrence at a lower PSA level. Therefore, imaging can potentially guide therapies. This may have implications to improve patient selection and volume delineation. Furthermore, any PET avid nodes could be simultaneously boosted to a higher dose. This would allow a more personalized approach, potentially improving RT outcomes and reducing toxicity. This further supports the combined high-dose salvage pelvic RT and ADT in the OLIGOPELVIS GETUG P07 trial that showed improved tumor control in oligo-recurrent pelvic node relapses with limited toxicity (31).

Finally, we observed an excellent in field control (93%) with only 10 patients having in-field recurrence and one having disease next to PLN RT volume. Similar rates of 88%–96% were reported in other studies (21, 24, 29, 30). These data show high efficacy of RT on local disease, and as such, further dose escalation is unlikely to improve local control. Moreover, recent data demonstrated that dose escalation (72 Gy) was not associated with PFS improvement compared with 66 Gy regimen (32). Ablative treatment of local recurrence in the PB after RP followed by RT is also an attractive strategy but, may generate significant toxicity rates and prospective studies are needed to evaluate its effectiveness (33).

High ISUP grade, seminal vesical invasion (pT3b stage), positive LN, and a short interval to BCR after RP seems to be the main factors which have a negative impact on disease progression, in line with previously published studies following primary treatment for PC (34). In our cohort, PSADT before RT did not differ between each group. However, the high proportion of missing PSA data before RT (23%) provides limited information and can be substantial bias.

Our study has several limitations. First, our present analyses are limited by their retrospective design and clinical practice evolution over the last 10 years, particularly with respect to RT techniques and imaging. PET-CT use, which increases sensitivity in recurrence detecting, was not systematic. As such, recurrence rate is also likely underreported, even if only 13 patients had clinically detected recurrences through CT imaging with a median PSA level of 2.5 ng/ml. Furthermore, some patients have a long interval of BCR before imaging (up to 86 months) which may impact patterns of recurrence. Finally, histological confirmation of PC relapses was obtained in only 11% of our cohort. Despite these limitations, we maintain that this study provides useful data on recurrence patterns following postoperative RT.

## Conclusion

Anatomic distribution of recurrence sites is consistent with previous imaging studies focused on BF after postoperative RT. While local RT failures are rare, patients remain at risk of metastatic progression. Pelvic disease is also a common site of failure, especially in men receiving postoperative RT to the PB alone. Further studies examining imaging directed salvage treatment are needed to personalize radiation therapy plan.

## Data Availability Statement

The original contributions presented in the study are included in the article/supplementary material. Further inquiries can be directed to the corresponding author.

## Ethics Statement

The studies involving human participants were reviewed and approved by research ethics committee (2019/88). The patients/participants provided their written informed consent to participate in this study. Written informed consent was obtained from the individual(s) for the publication of any potentially identifiable images or data included in this article.

## Author Contributions

AG-M, GC, and VS were responsible for the study design, analysis, and interpretation. AG-M and TP were responsible for data acquisition. AG-M drafted the article. All authors contributed to the article and approved the submitted version.

## Conflict of Interest

The authors declare that the research was conducted in the absence of any commercial or financial relationships that could be construed as a potential conflict of interest.

## Publisher’s Note

All claims expressed in this article are solely those of the authors and do not necessarily represent those of their affiliated organizations, or those of the publisher, the editors and the reviewers. Any product that may be evaluated in this article, or claim that may be made by its manufacturer, is not guaranteed or endorsed by the publisher.
